# Positive Margin Rates After Breast-Conserving Surgery by Histologic Subtype: A Systematic Review and Meta-analysis Evaluating the Impact of Oncoplastic Surgery

**DOI:** 10.1245/s10434-025-17329-2

**Published:** 2025-04-24

**Authors:** Kayla M. Switalla, Israel O. Falade, Astrid Quirarte, Molly Baxter, Mandeep Kaur, Rita A. Sakr, Giovanni Corso, Rita A. Mukhtar

**Affiliations:** 1 Department of Surgery, UCSF Breast Care Center, University of California San Francisco, San Francisco, CA USA; 2 University of Minnesota Medical School, Minneapolis, MN USA; 3 School of Medicine, University of California San Francisco, San Francisco, CA USA; 4 Department of Breast Oncoplastic Surgery, Emirates Hospital Group, Dubai, United Arab Emirates; 5 Department of Oncology and Hemato-Oncology, University of Milan, Milan, Italy; 6 Division of Breast Surgery, European Institute of Oncology (IEO), Istituto di Ricovero e Cura a Carattere Scientifico (IRCCS), Milan, Italy

**Keywords:** Oncoplastic surgery, Breast-conserving surgery (BCS), Positive margins, Invasive lobular carcinoma, Meta-analysis

## Abstract

**Background:**

Invasive lobular carcinoma (ILC), the second most common histologic subtype of breast cancer, has a higher risk of positive surgical margins than invasive ductal carcinoma (IDC). Whether this risk persists for patients undergoing breast-conserving surgery (BCS) with oncoplastic approaches remains unclear. We conducted a systematic review and meta-analysis to assess positive margins following oncoplastic BCS by histologic subtype and evaluate the impact of oncoplastic surgery on positive margins in ILC.

**Methods:**

We systematically searched the literature for articles reporting positive margin rates after oncoplastic BCS in ILC patients. Relative risks (RR) were log transformed and displayed with forest plots.

**Results:**

Eight studies, encompassing 754 ILC patients undergoing BCS (338 with oncoplastic surgery), were included. The pooled positive margin rate for ILC patients undergoing oncoplastic surgery was 31% (95% confidence interval [CI] 21–40%). Patients with ILC had a significantly higher RR for positive margins after oncoplastic BCS compared with IDC (RR 3.4, 95% CI 1.5–7.4). However, for ILC patients with larger tumors, oncoplastic BCS was associated with a significantly lower RR for positive margins compared with standard BCS (RR 0.5, 95% CI 0.3–0.9).

**Conclusions:**

Invasive lobular carcinoma patients undergoing oncoplastic BCS have higher positive margin risks than IDC patients, underscoring the need for improved preoperative imaging and systemic therapies. However, the addition of oncoplastic surgery to BCS reduces positive margin rates compared with standard BCS in ILC patients, particularly for larger tumors. These findings highlight the role of oncoplastic surgery as an important technique to optimize outcomes for those at high risk of positive margins.

**Supplementary Information:**

The online version contains supplementary material available at 10.1245/s10434-025-17329-2.

Breast-conserving surgery (BCS) with adjuvant radiation has been shown to have comparable recurrence and survival rates to that of total mastectomy, shifting the focus of surgical management towards techniques that prioritize both aesthetic outcomes and oncological safety.^1–5^ This evolution has led to the rise of oncoplastic BCS, an approach that combines volume displacement or replacement techniques to ensure thorough tumor resection while optimizing aesthetic results.^6,7^ Oncoplastic BCS frequently includes contralateral breast surgery to maintain symmetry, especially in procedures such as oncoplastic breast reduction, which combines partial mastectomy with bilateral reduction mammoplasty, thereby allowing surgeons to remove a larger volume of breast tissue with wider margins.^7–10^ Consequently, oncoplastic BCS can significantly enhance aesthetic and quality of life outcomes while potentially reducing the incidence of positive margins.^11–20^

A particularly pertinent application of oncoplastic surgery is in the treatment of invasive lobular carcinoma (ILC), the second most common histologic subtype of breast cancer.^21,22^ Characterized by its diffuse growth pattern, ILC presents unique challenges in clinical and radiological detection, as well as surgical management.^22,23^ Historically, patients with ILC undergoing BCS have faced a higher risk of positive surgical margins compared with the more common invasive ductal carcinoma (IDC).^24–26^ However, it is unclear whether this remains true for patients who undergo BCS with oncoplastic surgery.

Several studies have presented systematic analyses of positive margin rates following oncoplastic BCS for breast cancer in general; however, none have comprehensively examined this outcome specifically for patients with ILC.^27–31^ Given the limited data evaluating the benefit of oncoplastic surgery in ILC specifically, we conducted a systematic review and meta-analysis to assess overall positive margin rates after oncoplastic BCS in ILC patients. We also compared positive margin rates following oncoplastic BCS by histologic subtype, as well as positive margin rates in ILC patients by BCS type (oncoplastic BCS vs. standard BCS). This review aims to clarify the oncologic outcomes of oncoplastic BCS in ILC and better inform surgical strategies for the management of this particular subtype.

## Methods

### Study Design and Search Strategy

This review was conducted in accordance with Preferred Reporting Items for Systematic Reviews and Meta-Analyses (PRISMA) guidelines for meta-analyses reporting.^32,33^ A systematic search of the literature was conducted using PubMed, Embase, and Web of Science databases using a search strategy that was finalized with a clinical librarian (Supplementary Table 1). The databases were searched systematically by two independent authors for eligible studies. The search was completed in February 2024.

### Eligibility Criteria and Study Selection

We considered all studies that met the following criteria: (1) population: women with invasive breast cancer and histologic subtype specified, including some or all patients having ILC; (2) intervention: oncoplastic BCS for breast cancer; (3) outcome: positive margin rate after oncoplastic BCS in ILC patients; (4) study design: randomized clinical trials (RCTs), cohort, and case–control studies.

**Fig. 1:**
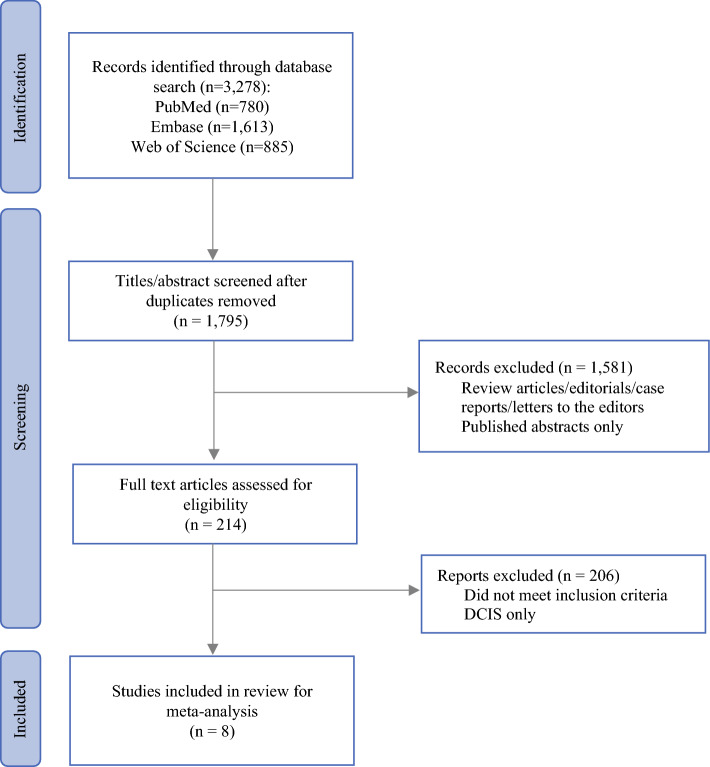
PRISMA flow chart of the literature screening process. *DCIS* ductal carcinoma in situ

Endnote software was used to remove duplicate studies, and the retrieved references were subsequently imported into a data collection tool to screen for relevance. Two authors initially reviewed titles and abstracts for relevance, followed by full text reviews (Fig. 1). Where multiple studies reported on the same group of patients, data were included in the analysis once only. Any disagreements were resolved by a third reviewer. Review articles, editorials, case reports, letters to the editors, published abstracts only, or reports on DCIS only were excluded.

### Data Extraction

Once the included studies were finalized, two independent authors extracted data on study design including the following elements: retrospective versus prospective design, country, year of publication, number of patients with ILC undergoing standard BCS, number of patients with ILC/IDC undergoing oncoplastic BCS, type of oncoplastic BCS, patient age (mean/median and range), tumor size (mean/median and range), and neoadjuvant therapy use. Any disagreements were solved by group discussion.

### Definitions of Primary and Secondary Outcomes

The primary outcome of our study was positive margin rates after oncoplastic BCS in ILC patients, which was defined as the proportion of ILC patients who had positive histological margins after oncoplastic BCS. Positive margins were defined as “ink on tumor,” tumor <1 mm from ink margin, or tumor <10 mm from ink margin, according to the definition utilized by each study (Table 1).

**Table 1:** Descriptive characteristics of studies included in the meta-analysis

Author, year	Country, journal	Type of study	Study date range	No. patients undergoing oncoplastic surgery		No. ILC patients undergoing standard BCS	Exclusion criteria	Type of oncoplastic surgery	Definition of positive margins
				ILC	IDC				
Sakr, 2011	France, *European Journal of Surgical Oncology*	Retrospective (cohort)	2005–2008	26	n/a	47		Level I or Level II OPS	a
Grubnik, 2013	South Africa, *World Journal of Surgery*	Retrospective (cohort)	2002–2009	7	202	n/a		Therapeutic mammoplasty	b
Ho, 2016	Scotland (UK), *Breast Cancer-Basic and Clinical Research*	Prospective (cohort)	2010–2015	5	23	n/a	Previous DCIS or breast cancer	Volume replacement OBCS	c
Clough, 2018	France, *Annals of Surgical Oncology*	Prospective (cohort)	2004–2016	43	239	n/a		Level II mammoplasties	d
Palsdottir, 2018	Iceland, *Scandinavian Journal of Surgery*	Retrospective (cohort)	2008–2014	10	61	50	Mastectomy, no tumor seen in the removed breast tissue, bilateral SBCS, and males.	Volume displacement and volume replacement procedures	nd
Romics, 2018	Scotland (UK), *European Journal of Surgical Oncology*	Retrospective (cohort)	2005–2017	53	413	n/a		Level II OBCS	c
Crown, 2021	USA, *Annals of Surgical Oncology*	Retrospective (cohort)	2012–2018	27	73	n/a	Pure DCIS	Comprehensive OBCS	e
Falade, 2024	USA, *Annals of Surgical Oncology*	Retrospective (cohort)	1995–2023	167	n/a	319		Level I or Level II OBCS	d

*ILC* invasive lobular carcinoma; *IDC* invasive ductal carcinoma; *DCIS* ductal carcinoma in situ; *OPS* oncoplastic surgery; *OBCS* oncoplastic breast-conserving surgery; *SBCS * standard breast-conserving surgery; *n/a* not applicable; *nd* not defined

Comprehensive Oncoplastic Breast Conservation Surgery: Including operations, such as the Modified Radial Ellipse, Mastopexy, Racquet Mammoplasty, and Reduction Mammoplasty, including Neoareolar Reduction Mammoplasty with Immediate Nipple Reconstruction

Definition of positive margins: (a) margin status was considered positive if invasive carcinoma was found at ink on the edge of resected specimen and within 1 mm; or close if it was <2 mm on final histologic review; (b) close margins (<10 mm) and a minimum margin of 10 mm was deemed acceptable; (c) negative if least 1 mm between cut edge of the specimen and the outer limit of the tumor; (d) positive margins were defined as “ink on the tumor”; (e) before 2014, margins were considered adequate if they were ≥2 mm, for both invasive cancer and DCIS. Following 2014, “no tumor on ink” was considered an adequate margin

Additionally, we documented several secondary outcomes from studies that provided relevant data. These outcomes included comparison of positive margin rates by oncoplastic BCS versus standard BCS in patients with ILC and comparison of positive margin rates in ILC versus IDC following oncoplastic BCS. Finally, we assessed the rate of successful oncoplastic BCS in patients with ILC, which was defined as the proportion of ILC patients who did not require completion mastectomy, as well as the rate of locoregional or distant recurrence after oncoplastic BCS in ILC when reported.

### Evaluation of Methodological Quality and Risk of Bias

We utilized the Newcastle-Ottawa scale (NOS) to evaluate the risk of bias in our included studies. The NOS is a validated and widely used tool for evaluating the quality of nonrandomized studies across three domains: selection, comparability, and outcome (cohort studies) (Supplementary Methods 1). The scale also categorizes studies into three quality levels based on their scores: low quality (0–3 stars), indicating significant risk of bias and methodological limitations; moderate quality (4–6 stars), reflecting some concerns regarding bias and confounding; and high quality (7–9 stars), denoting minimal bias, well-controlled confounders, and reliable outcome measures.^34^ The quality of eligible articles was independently assessed by two investigators, and any disagreements were resolved by the third investigator.

### Statistical Analyses

We gathered study characteristics and patient information from all studies included in the meta-analysis. Positive margin rates after oncoplastic BCS in ILC patients were compiled from individual studies to obtain an overall pooled positive margin estimate. We then conducted a meta-analysis comparing positive margin rates after oncoplastic BCS by histologic subtype (ILC vs. IDC). We conducted an additional meta-analysis comparing positive margin rates in ILC patients by BCS type (oncoplastic BCS vs. standard BCS). Finally, to understand the impact of tumor size on positive margin rates after oncoplastic BCS, an unplanned subset meta-analysis was performed comparing positive margin rates by BCS type specifically in ILC patients with T3 tumors when individual tumor size was available. Patient-level data were available for one study (Falade, 2024); therefore, this subset meta-analysis was performed, including patients with T3 tumors only from Falade et al.’s study, aiming to discern any potential benefits within this subgroup of patients with larger tumors.

Relative risk (RR) ratios were used to compare the relative risk of positive margins between groups. Relative risk was calculated for each included study by using the proportion of positive margins in the ILC oncoplastic BCS group divided by the proportion of positive margins in either the ILC standard BCS group or the IDC oncoplastic BCS group. Standard errors (SE) for each RR estimate were calculated using an established formula.^35^ Logarithmic transformation was applied to both the RR and SE prior to analysis to address non-normality and reduce skewness of the data. The transformed SE was then used to calculate confidence intervals (CI) for each study in the meta-analysis. All meta-analyses were conducted by combining the logarithmic transformed data for each study and by using random-effects model (or fixed-effects model in the case of none or minimal heterogeneity). Heterogeneity was assessed across studies by using the I^2^ statistic; values greater than 50% indicated substantial heterogeneity. All statistical analyses was performed by using Stata version 18.0 (College Station, TX: StataCorp LLC). Results were considered statistically significant if the *p*-value was <0.05 or if the 95% CI did not include 1.00.

## Results

### Literature Search

Our initial literature search identified 3,278 references. After removal of duplicates and final screening, eight studies were included in the final systematic review and meta-analysis (Fig. 1).^36–43^ The eight studies encompassed a total of 1,765 patients. Of these, 338 were ILC patients in the oncoplastic BCS group, 416 were ILC patients in the standard BCS group, and 1,011 were IDC patients in the oncoplastic BCS group.

### Study Characteristics and Overall Positive Margin Rate

**Fig. 2:**
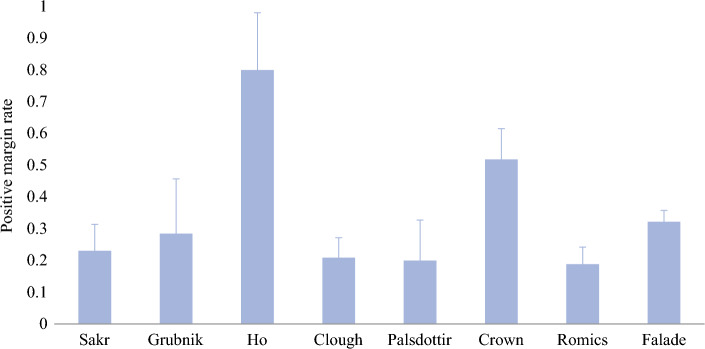
Positive margin rates after oncoplastic breast-conserving surgery in invasive lobular carcinoma patients (95% confidence intervals)

Among the eight studies included in our final analysis, six were retrospective reviews and two were prospective trials. The studies were conducted across five different countries (Table 1). Five studies compared positive margin rates after oncoplastic surgery in ILC patients versus IDC patients, whereas three studies compared positive margin rates in ILC patients after oncoplastic surgery versus standard BCS. Tables 1 and 2 summarize additional characteristics of the included studies. All included studies reported data on prevalence of positive margins in ILC patients undergoing oncoplastic BCS, with a pooled prevalence of 31% (95% CI 21–40%) (Fig. 2). Additional patient and tumor characteristics for each of the eight included studies are displayed in Supplementary Tables 2, 3, 4, 5, 6, 7, 8 and 9. Based on the NOS checklist, all included studies were categorized as high-quality (Table 3).

**Table 2:** Summary of patient and tumor characteristics of studies included in the meta-analysis

Author, year	Age (years)	Pathologic tumor size	Tumor grade, *n* (%)	Neoadjuvant therapy	Follow-up
Sakr, 2011	Overall (mean): 58 [range 37–76]	Overall (mean): 16 mm	n/a	n/a	N/A
Grubnik, 2013	Overall (mean): 56.3 [range 28–80]	Overall (mean): 15.4 mm	G1: 58 (23.9%) G2: 107 (44%) G3: 78 (32%)	NAC: 64/251 overall patients (25.5%)	50 months mean
Ho, 2016	Overall (mean): 51 [range 24–69]	Overall (mean): 25 mm	G1:1 (3.3%) G2:13 (43.3.%) G3:16 (53.3%)	NAC: 2/30 (6.7%)	48.5 months median
Clough, 2018	Overall (mean): 57 [median 58; range 20–86]	Overall length (mean): 26 mm Overall weight (mean): 177 g Overall volume (mean): 331 cm^3^	G1: 122 (14.5%) G2: 157 (55.7%) G3: 85 (29.8%)	NAC: 73/262 with invasive cancer (27.9%)	55 months median
Mukhtar, 2018	Overall (mean): 61 [range 28–97]	ILC OBCS (mean): 2.75 cm/129.5 cm^3^ ILC BCS (mean): 2.2 cm/65 cm^3^	G1: 122 (34%) G2: 223 (62%) G3: 14 (4%)	NAC: 33/106 (31.1%) NET: 36/242 (15%)	N/A
Palsdottir, 2018	BCS (median): 62 [range 28–94] OBCS (median): 50 [range 27–75]	BCS length/weight (median) = 1.5 cm/51.8 g OBCS length/weight (median) = 2 cm/126 g	n/a	n/a	N/A
Romics, 2018	Overall (median): 56 [range 21–86]	Overall (median) = 26 mm	G1: 50 (10.1%) G2: 243 (49.2%) G3: 197 (39.9%) Incomplete: 4 (0.8%)	Overall: 142/496 with invasive cancer (28.6%) NAC: 68/496 (13.7%) NET: 74/496 (14.9%)	30 months median
Crown, 2021	Overall (median): 59 [range 29–84]	81 patients with multifocal or multicentric disease (mean): 44.6 ± 36.5 mm for largest single tumor 19 patients with unifocal disease (mean): 78.7 ± 30.1 mm	G1: 13 (13%) G2: 50 (50%) G3: 37 (37%)	NAC: 8/100 (8%)	40 months median
Falade, 2024	Overall (mean): 61.4	Not reported	G1: 149 (30.8%) G2: 312 (64.6%) G3: 22 (4.6%)		N/A

*ILC* invasive lobular carcinoma; *BCS* breast-conserving surgery; *OBCS* oncoplastic breast-conserving surgery; *G1* grade 1; *G2* grade 2; *G3* grade 3; *N/A* not applicable; *NAC* neoadjuvant chemotherapy; *NET* neoadjuvant endocrine therapy

**Table 3:** Assessment of included studies utilizing the Newcastle-Ottawa quality assessment scale

Study, year	Selection*	Comparability**	Outcome^***^	Overall
Sakr, 2011	4	1	2	7/9
Grubnik, 2013	4	0	3	7/9
Ho, 2016	4	0	3	7/9
Clough, 2018	4	0	3	7/9
Palsdottir, 2018	4	2	2	8/9
Romics, 2018	4	0	3	7/9
Crown, 2021	4	0	3	7/9
Falade, 2024	4	1	3	8/9

^*^Maximum 4 stars

^**^Maximum 2 stars

^***^Maximum 3 stars

### Meta-analysis of Positive Margin Rates by Histologic Subtype

**Fig. 3:**
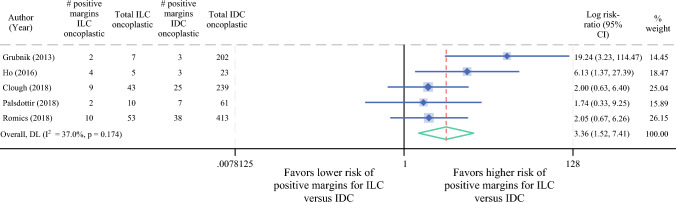
Meta-analysis comparing positive margins rates after oncoplastic surgery in ILC patients versus IDC patients. *ILC* invasive lobular carcinoma; *IDC* invasive ductal carcinoma

Five studies were included in our meta-analysis comparing positive margin rates after oncoplastic BCS in ILC versus IDC patients. Our results showed that lobular histology, compared with ductal histology, exhibited a significantly higher relative risk for positive margins after oncoplastic BCS (RR 3.4, 95% CI 1.5–7.4) (Fig. 3).

### Meta-analysis of Positive Margin Rates in ILC Patients by Type of BCS

**Fig. 4:**
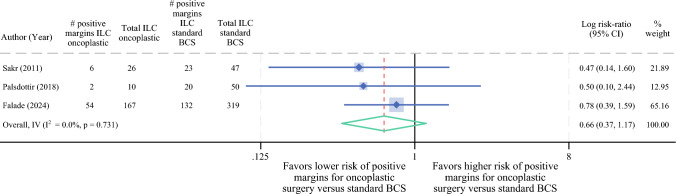
Meta-analysis comparing positive margin rates in ILC patients after oncoplastic BCS versus standard BCS. *ILC* invasive lobular carcinoma; *IDC* invasive ductal carcinoma; *BCS* breast-conserving surgery

**Fig. 5:**
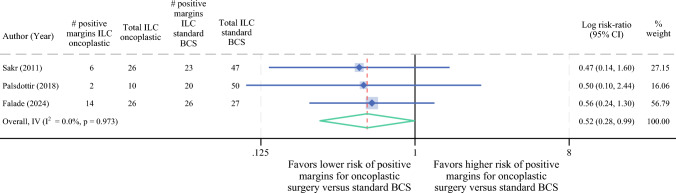
Subset meta-analysis comparing positive margin rates after oncoplastic BCS versus standard BCS in ILC patients with T3 tumors (when size was reported). *ILC* invasive lobular carcinoma; *IDC* invasive ductal carcinoma; *BCS* breast-conserving surgery

Three studies were included in our meta-analysis comparing positive margin rates in ILC patients undergoing oncoplastic BCS versus standard BCS. Oncoplastic BCS demonstrated a lower relative risk of positive margins compared to standard BCS (RR 0.7, 95% CI 0.4–1.2), although this did not reach statistical significance (Fig. 4). However, in our additional subset meta-analysis restricted to T3 tumors for Falade et al.’s study, oncoplastic BCS was associated with significantly lower risk for positive margins compared with standard BCS (RR 0.5, 95% CI 0.3–0.9) (Fig. 5).

### Recurrence Rates

Recurrence rates after oncoplastic BCS in ILC patients were reported in three studies (Ho, 2016; Clough, 2018; Falade, 2024). Ho et al. reported 0% locoregional recurrences over a median follow-up of 48.5 (range 6–66) months. Clough et al. reported a cumulative 5-year incidence of local and distant recurrence to be 0% and 11.5%, respectively, for patients with ILC undergoing oncoplastic BCS. Data from Falade et al. showed an overall recurrence rate (any local or distant recurrences) of 7.1% after oncoplastic BCS for ILC patients.

### Prevalence of Successful BCS

Rates of successful BCS after oncoplastic surgery in ILC patients were reported in two studies (Crown, 2021; Falade, 2024). Crown et al. reported a completion mastectomy rate of 11% (i.e., successful oncoplastic BCS rate of 89%), whereas Falade et al. reported a successful oncoplastic BCS rate of 91.2%.

## Discussion

Oncoplastic surgery for breast cancer has gained interest given its focus on both oncologic and aesthetic outcomes. This approach involves wide local excision of the tumor followed by reconstruction of the defect, thus reducing the likelihood of positive resection margins while providing a cosmetically acceptable result.^44,45^ However, to date, there is no comprehensive analysis of positive margin rates and other surgical outcomes after oncoplastic BCS specifically in ILC patients. Thus, we performed a systematic review and meta-analysis of the available literature with the goal to evaluate the utility of oncoplastic surgery for patients with ILC. The findings from our meta-analysis reaffirmed the higher positive margin rates in ILC patients compared with IDC patients. However, we also demonstrated a trend toward oncoplastic surgery resulting in lower positive margin rates for patients with ILC compared to standard BCS, especially in patients with larger tumors.

It is well established in the literature that breast cancer patients with lobular histology face an elevated risk of positive margins, especially compared to patients with ductal histology.^24–26^ Our analysis reaffirmed this, demonstrating that individuals with ILC had a threefold higher relative risk of positive margins compared to those with IDC, even after undergoing oncoplastic BCS. The elevated risk associated with ILC can be attributed, in part, to its diffuse growth nature, which poses a challenge for standard imaging modalities in accurately reflecting ILC tumor size, leading to higher stage at presentation.^23,46–48^ This suggests that relying solely on oncoplastic techniques may not sufficiently address the disparity in positive margin rates between ILC and IDC cases.

To address these challenges, there is potential in integrating new imaging techniques alongside oncoplastic approaches. Our institution, along with many others, have been exploring novel imaging techniques specifically tailored for ILC tumors, such as dedicated breast PET/CT and contrast-enhanced mammography.^23,49–51^ By combining these advanced imaging modalities with novel surgical techniques, such as oncoplastic surgery, the goal is to enhance preoperative radiographic detection and subsequently improve surgical outcomes for ILC cases. Importantly, although patients with ILC have higher risk of positive margins than those with IDC even after oncoplastic surgery, single-institution data suggest that this increased risk does not translate to worse oncologic outcomes.^43^ While reexcision may be required, long-term recurrence risk is similar for patients with ILC undergoing immediate oncoplastic surgery versus standard BCS.^43^

Despite this clearly elevated risk of positive margins for ILC, our findings also showed an interesting trend in outcomes when utilizing oncoplastic BCS. Our pooled positive margin rate for ILC patients undergoing oncoplastic surgery was 31%, which is lower than the reported range of positive margin rates for ILC patients undergoing standard BCS, which ranges on the higher end of 35–88%.^52–56^ Indeed, compared with ILC patients undergoing standard BCS, our findings also demonstrated that ILC patients undergoing oncoplastic BCS experienced lower risks for positive margins, although this trend did not reach statistical significance in our overall cohort. Of note, one limitation of this meta-analysis is the variability in the definition of positive margins across studies. While “no ink on tumor” is accepted as negative margins by Society of Surgical Oncology guidelines, some contributing studies required wider margins. How this difference in positive margin definition impacted our results is unknown, but it is plausible that oncoplastic surgery would have resulted in a higher rate of negative margins in studies with a less stringent margin definition.

Interestingly, when restricting the largest study of this meta-analysis to T3 patients only, the advantage of oncoplastic surgery in lowering positive margins became evident and statistically significant. While the use of shave margins was beyond the scope of this review, we note that a high proportion of patients in the Falade et al. study did indeed have shave margins taken—a technique previously shown to reduce positive margin rates after BCS.^57^ Moreover, our institution has previously shown that the use of shave margins can be beneficial for mitigating positive margin risk specifically for ILC tumors.^20^ Thus, for patients with smaller tumors, incorporating oncoplastic surgery in addition to shave margins may offer limited additional benefit.

Conversely, for patients with larger tumors, shave margins alone may not suffice to reduce the risk of positive margins, making oncoplastic surgery particularly beneficial. Indeed, several prospective and retrospective studies have highlighted the advantage of oncoplastic surgery, particularly in achieving safe BCS in patients with larger tumors, termed “extreme oncoplasty.”^58–61^

To our knowledge, this is the first comprehensive literature review and meta-analysis to compare positive margin rates after oncoplastic BCS in patients with ILC, evaluating a large sample of more than 1,000 patients across five different countries. However, there were several limitations to our study. Given the lack of published data surrounding this topic, we were limited by the number of studies and the sample sizes included in our meta-analysis, with our own institutional study having the largest sample size. Additionally, the majority of studies that were included were observational in nature. Most studies did not perform multivariable analyses for our outcome of interest and therefore, without patient-level data available, we were unable to account for confounding variables that may influence surgical outcomes, such as the ratio of tumor size to breast size, use of shave margins, nodal status, tumor receptor status, and neoadjuvant and/or adjuvant therapies. Finally, our analyses were limited by the variability in oncoplastic techniques and definitions of positive margins, as studies were performed in different years and followed the oncological guidelines appropriate at the time. Nonetheless, our cumulative data suggest a benefit of oncoplastic surgery for ILC patients, underscoring the need for further research on oncoplastic techniques to enhance surgical outcomes in this high-risk population.

## Conclusions

Oncoplastic BCS is an evolving surgical technique that prioritizes thorough tumor resection while optimizing aesthetic results. Our systematic review and meta-analysis demonstrated a persistently elevated risk of positive margins in ILC patients compared with IDC patients, even after undergoing oncoplastic BCS. Yet, we also showed a trend towards a lower risk of positive margins in ILC patients after oncoplastic BCS compared with standard BCS, especially in patients with larger tumor sizes. These findings suggest that the benefit of oncoplastic surgery is not limited to a single institution and should be generalizable to the majority of ILC patients. Our results also underscore the need for further research on oncoplastic techniques to improve surgical outcomes for this population at high risk of positive margins.

## Supplementary Information

Below is the link to the electronic supplementary material.

Supplementary file1 (DOCX 16 KB)

Supplementary file2 (DOCX 51 KB)
