# Supplementary material for: Positive Margin Rates After Breast-Conserving Surgery by Histologic Subtype: A Systematic Review and Meta-analysis Evaluating the Impact of Oncoplastic Surgery
Source: Ann Surg Oncol. 2025 Apr 24;32(7):4899–909. doi: 10.1245/s10434-025-17329-2 (PMC12129873; doi:10.1245/s10434-025-17329-2)
Supplement: Supplementary file 1 — Supplementary file1 (DOCX 16 KB) [file 10434_2025_17329_MOESM1_ESM.docx]

**Supplementary Methods 1:** The Newcastle Ottawa Quality Assessment Scale for cohort studies

Note: A study can be awarded a maximum of one star for each numbered item within the Selection and Outcome categories. A maximum of two stars can be given for Comparability

Selection

**1) Representativeness of the exposed cohort**

a) truly representative of the average _______________ (describe) in the community ☆

b) somewhat representative of the average ______________ in the community ☆

c) selected group of users eg nurses, volunteers

d) no description of the derivation of the cohort

**2) Selection of the non-exposed cohort**

a) drawn from the same community as the exposed cohort ☆

b) drawn from a different source

c) no description of the derivation of the non-exposed cohort

**3) Ascertainment of exposure**

a) secure record (eg surgical records) ☆

b) structured interview ☆

c) written self-report

d) no description

**4) Demonstration that outcome of interest was not present at start of study**

a) yes ☆

b) no

Comparability

**1) Comparability of cohorts on the basis of the design or analysis**

a) study controls for _____________ (select the most important factor) ☆

b) study controls for any additional factor (This criteria could be modified to indicate specific controls for a second important factor.) ☆

Outcome

**1) Assessment of outcome**

a) independent blind assessment ☆

b) record linkage ☆

c) self-report

d) no description

**2) Was follow-up long enough for outcomes to occur**

a) yes (select an adequate follow up period for outcome of interest) ☆

b) no

**3) Adequacy of follow up of cohorts**

a) complete follow up - all subjects accounted for ☆

b) subjects lost to follow up unlikely to introduce bias - small number lost - > ____ % (select an adequate %) follow up, or description provided of those lost) ☆

c) follow up rate < ____% (select an adequate %) and no description of those lost

d) no statement
