# Supplementary material for: Positive Margin Rates After Breast-Conserving Surgery by Histologic Subtype: A Systematic Review and Meta-analysis Evaluating the Impact of Oncoplastic Surgery
Source: Ann Surg Oncol. 2025 Apr 24;32(7):4899–909. doi: 10.1245/s10434-025-17329-2 (PMC12129873; doi:10.1245/s10434-025-17329-2)
Supplement: Supplementary file 2 — Supplementary file2 (DOCX 51 KB) [file 10434_2025_17329_MOESM2_ESM.docx]

**Supplementary Table 1.** Database Search Strategies

| **Database** | **Search Strategy** |
| --- | --- |
| PubMed | breast[tiab] AND (Oncoplastic Surgery OR oncoplastic closure OR oncoplastic reconstruction OR extreme oncoplasty OR extreme oncoplastic surgery OR oncoplastic technique OR oncoplastic reduction mammoplasty OR oncoplastic breast-conserving surgery) AND (outcome* OR recurr* OR positive margins OR complications OR survival OR morbidity OR mortality OR safety OR risk*) NOT review |
| Embase | ('oncoplastic surgery'/exp OR 'oncoplastic surgery' OR (oncoplastic AND ('surgery'/exp OR surgery)) OR 'oncoplastic closure' OR (oncoplastic AND closure) OR 'oncoplastic reconstruction' OR (oncoplastic AND ('reconstruction'/exp OR reconstruction)) OR 'extreme oncoplasty' OR (extreme AND oncoplasty) OR 'extreme oncoplastic surgery' OR (extreme AND oncoplastic AND ('surgery'/exp OR surgery)) OR 'oncoplastic technique' OR (oncoplastic AND ('technique'/exp OR technique)) OR 'oncoplastic reduction mammoplasty' OR (oncoplastic AND ('reduction'/exp OR reduction) AND ('mammoplasty'/exp OR mammoplasty)) OR 'oncoplastic breast-conserving surgery' OR (oncoplastic AND 'breast conserving' AND ('surgery'/exp OR surgery))) AND (outcome* OR recurr* OR 'positive margins' OR (positive AND margins) OR 'complications'/exp OR complications OR 'survival'/exp OR survival OR 'morbidity'/exp OR morbidity OR 'mortality'/exp OR mortality OR 'safety'/exp OR safety OR risk*) AND breast:ab,ti NOT review:ab,ti |
| Web of Science | Results for (Oncoplastic Surgery OR oncoplastic closure OR oncoplastic reconstruction OR extreme oncoplasty OR extreme oncoplastic surgery OR oncoplastic technique OR oncoplastic reduction mammoplasty OR oncoplastic breast-conserving surgery) AND (outcome* OR recurr* OR positive margins OR complications OR survival OR morbidity OR mortality OR safety OR risk*) (All Fields) AND breast (Abstract) NOT review (Title) and Article or Early Access (Document Types) |

**Supplementary Table 2.** Original Table 1 from Sakr et al. (2011).

Table 1: Univariate analysis of the 73 patients with BCS and ILC

| **Characteristic** | **Negative margins**  **(N=44)** | **Close/positive margins**  **(N=29)** | ***p-value*** |
| --- | --- | --- | --- |
| Mean age (years, range) | 59 (41-76) | 55 (37-74) | <0.039 |
| Post-menopausal status (n,%) | 33 (75) | 19 (65) | ns |
| Familial history of breast  cancer (n) | 10 (23) | 3 (10) | ns |
| History of HRT (n) | 8 (18) | 2(7) | ns |
| Tumor imaging size (mean, mm) | 13 (4-30) | 13 (4-20) | ns |
| Palpable mass (n) | 26 (59) | 18 (62) | ns |
| Specimen resection size (mean, mm) | 63 (30-110) | 60 (30-90) | ns |
| Tumor histology size (mean, mm) | 15 (2-47) | 23 (10-50) | 0.002 |
| Tumor stage |  |  | 0.006 |
| T1 (n) | 33 (75) | 12 (41) |  |
| T2 (n) | 11 (25) | 17 (59) |  |
| Multifocality (n) | 7 (16) | 24 (83) | 0.000 |
| Grade 1/2 (n) | 40 (91) | 28 (96) | ns |
| Low mitotic index (n) | 35 (79) | 22 (76) | ns |
| ER positive (n) | 43 (98) | 27 (93) | ns |
| PR positive (n) | 31 (70) | 23 (79) | ns |
| Tumor location |  |  |  |
| Upper outer | 22 (50) | 14 (48) | ns |
| Upper inner | 10 (23) | 15 (52) | 0.013 |
| Lower | 10 (23) | 0 | - |
| Central | 2 (5) | 0 | - |
| Surgery type |  |  | 0.045 |
| FTE | 24 (55) | 23 (79) |  |
| OPS | 20 (45) | 6 (21) |  |

BCS: breast conservative surgery; ILC: invasive lobular carcinoma; HRT: hormonal replacement therapy, ns: not significant.

**Supplementary Table 3.** Recreated table of patient and tumor characteristics using original results from Grubnik et al. (2013)

Table: Patient and tumor characteristics

|  | No. of patients (%) |
| --- | --- |
| Age, mean (range) | 56.3 (28-80) |
| Menopausal status |  |
| Pre-menopausal | 62 (24.7) |
| Menopausal | 189 (75.3) |
| Histology |  |
| Invasive ductal Ca | 131 (52.2) |
| Invasive ductal Ca with DCIS | 71 (28.3) |
| Invasive lobular Ca | 6 (2.4) |
| Mixed ductal lobular | 1 (0.4) |
| Mixed ductal tubular | 1 (0.4) |
| Colloid/mucinous | 7 (2.8) |
| Tubular | 2 (0.8) |
| Tubulo-lobular | 2 (0.8) |
| Micropapillary | 1 (0.4) |
| Metaplastic | 2 (0.8) |
| Paget's disease of the nipple | 1 (0.4) |
| DCIS | 26 (10.3) |
| Tumor staging at presentation |  |
| Tis | 25 (10) |
| T1a | 5 (2) |
| T1b | 47 (19) |
| T1c | 90 (36) |
| T2 | 73 (29) |
| T3 | 3 (1) |
| T4 | 8 (3) |
| Underwent hormone replacement therapy | 140 (55.8) |
| Underwent neoadjuvant chemotherapy | 64 (25.5) |
|  |  |
|  |  |
|  |  |

**Supplementary Table 4.** Original Table 1 from Ho et al. (2016).

Table 1. Baseline characteristics and risk factors.

| VARIABLE | (n, %) |
| --- | --- |
| Age (mean, range) | 51, 24-69 |
| BMI (mean, range) | 27.8, 23.6-36.2 |
| Diabetes | |
| Yes | 1 |
| No | 24 |
| No data | 1 |
| Family history | |
| Yes | 5 |
| No | 21 |
| Smoking status | |
| Current smoker | 6 |
| Ex-smoker | 2 |
| Non-smoker | 20 |
| HRT | |
| Yes | 4 |
| No | 20 |
| No data | 2 |
| Immunosuppression |  |
| Yes | 0 |
| No | 30 |
| Breast cup size |  |
| A | 3 |
| B | 4 |
| C | 4 |
| D | 3 |
| E | 2 |
| F | 2 |
| Larger than F | 3 |
| No data | 7 |

HRT: hormone replacement therapy

**Supplementary Table 5.** Original Table 1 from Clough et al. (2018).

TABLE 1. Patient and Tumor Characteristics

| **Characteristic** | **N** | **%** |
| --- | --- | --- |
| Mean Age, yrs | 57 (median 58, range 20-86) |  |
| Mean radiological tumor size, mm | 28.7 (median 25, range 4- 150) |  |
| Mean histological tumor size, mm | 26 (median 20, range 0-180) |  |
| Focality |  |  |
| Unifocal | 292 | 83.4 |
| Multifocal | 58 | 16.6 |
| Neoadjuvant therapy |  |  |
| Yes | 73 | 27.9* |
| No | 189 | 72.1 |
| Pathological T stage |  |  |
| pTis | 68 | 19.4 |
| pTl | 155 | 44.3 |
| pT2 | 109 | 31.1 |
| pT3 | 18 | 5.1 |
| Histology |  |  |
| Pure ductal carcinoma in situ | 68 | 19.4 |
| Invasive ductal carcinoma | 239 | 68.3 |
| Invasive lobular carcinoma | 43 | 12.3 |
| Histologic subtype (invasive) |  |  |
| Luminal A | 166 | 63.4 |
| Luminal B (Her2 negative) | 3 | 1.1 |
| Luminal B (Her2 positive) | 17 | 6.5 |
| Her2 non luminal | 12 | 4.6 |
| Triple negative breast cancer | 62 | 23.7 |
| Missing | 2 | 0.8 |
| SBR grade |  |  |
| I | 41 | 14.5 |
| II | 157 | 55.7 |
| III | 84 | 29.8 |
| Mean specimen weight, g | 177 (median 127, range 40-1540) |  |
| Mean specimen volume, cm? | 331 (median 237, range 30-4031) |  |
| Margins | | |
| Clear | 306 | 87.4 |
| Involved | 44 | 12.6 |
| Nodal status | | |
| N0 | 248 | 70.9 |
| NI | 76 | 21.7 |
| N2 | 26 | 7.4 |
| Adjuvant chemotherapy | | |
| Yes | 111 | 31.7 |
| No | 239 | 68.3 |
| Adjuvant hormonotherapy | | |
| Yes | 240 | 68.6 |
| No | 110 | 31.4 |

*Calculated over 262 invasive cancers.

**Supplementary Table 6.** Original Table 1 from Palsdottir et al. (2018).

TABLE 1

Demographic characteristics.

|  | SBCS (n =665) | OBCS (n= 85) | P-value |
| --- | --- | --- | --- |
| Age. Median (range), years | 62.0 (28-94) | 50.0 (27-75) | <0.001 |
| Size of tumor. Median (range), cm | 1.5 (0.1-5.5) | 2.0 (0.4-8.0) | <0.001 |
| Weight of breast tissue. Median (range), g | 51.8 (1.8-660) | 126.0 (23.5-1010) | <0.001 |
| T and N stages | | | |
| T0, n (%) | 0 (0) | 0 (0) |  |
| T1, n (%) | 429 (64.5) | 33 (38.8) | <0.001 |
| T2, n (%) | 202 (30.3) | 43 (50.6) | <0.001 |
| T3, n (%) | 35 (5.2) | 7 (8.2) | 0.383 |
| N0, n (%) | 400 (60.1) | 49 (57.6) | 0.745 |
| N1, n (%) | 126 (18.9) | 20 (23.5) | <0.001 |
| N2, n (%) | 13 (2.0) | 0 (0) | 0.390 |
| N3, n (%) | 9 (1.4) | 4 (4.7) | 0.073 |
| Smoking, n (%) | 152 (22.8) | 6 (7.1) | <0.05 |
| Hormone therapy, n (%) | 141 (21.2) | 7 (8.2) | <0.05 |
| Admission time. Median (range), days | 1 (0.5-17) | 2 (1.0-5) | <0.001 |
| Positive family history, n (%) | 104 (12.9) | 11 (15.6) | 0.624 |
| DCIS, n (%) | 48 (7.2) | 9 (10.6) | 9.375 |
| Positive margins, n (%) | 133 (20.0) | 12 (14.3) | 0.251 |
| Complications, n (%) | 73 (11.0) | 11 (12.0) | 0.720 |
| Reoperations, n (%) | 91 (13.6) | 12 (14.1) | 1.000 |
| Hematoma | 11 (1.7) | 0 (0) |  |
| Extended wedge | 21 (3.2) | 1 (1.2) |  |
| Mastectomia | 52 (7.8) | 10 (11.8) |  |
| Necrosis | 5 (0.8) | 0 (0) |  |
| Node dissection | 2 (0.3) | 1 (1.2) |  |
| Time between surgery and adjuvant treatment. Median (range), days | 50.0 (15-202) | 47.5 (22-111) | 0.05 |

OBCS: oncoplastic breast-conservation surgery; SBCS: standard breast-conservation surgery; DCIS: ductal carcinoma in situ.

**Supplementary Table 7.** Original Table 5 from Romics et al. (2018).

Table 5: Tumour characteristics.

|  | *All patients* | *Patients ^a^ with 5-year follow-up* |
| --- | --- | --- |
| **Histological type** | **n = (%)** | **n= (%)** |
| Ductal | 413 (70.2%) | 182 (70.3%) |
| Lobular | 53 (9%) | 25 (9.6%) |
| Mixed ductal and lobular | 6 (1%) | 2 (0.8%) |
| Mixed ductal and papillary | 1 (0.2%) |  |
| Tubular | 7 (1.2%) | 4 (1.5%) |
| Mucinous | 6 (1%) | 4 (1.5%) |
| Metaplastic | 2 (0.3%) | 1 (0.4%) |
| Not determined (CPR) | 5 (0.8%) | 1 (0.4%) |
| DCIS | 78 (13.2%) | 36 (13.9%) |
| Paget's disease | 2 (0.3%) | 2 (0.8%) |
| Papillary carcinoma | 1 (0.2%) | 1 (0.4%) |
| LCIS | 3 (0.5%) | 1 (0.4%) |
| Hamartoma | 1 (0.2%) | n/a |
| Phyllodes | 8 (1.3%) | n/a |
| Diabetic mastopathy | 1 (0.2%) | n/a |
| Basal cell carcinoma | 1 (0.2%) | n/a |
| Osteosarcoma | 1 (0.2%) | n/a |
| Total | 589 (100%) | 259 (100%) |
| **Pathological t stage** |  |  |
| Tis | 83 (14.4%) | 39 (15%) |
| - [ypTO] | 13 (2.2%) [13] | 5 (1.9%) [5] |
| Tla [ypT1a] | 18 (3.2%) [6] | 17 (6.6%) [4] |
| T1b [ypT1b] | 47 (8.1%) [10] | 28 (10.9%) [4] |
| T1c lypT1c\| | 142 (24.6%) [30] | 33 (12.7%) [11] |
| 12 lyp12\| | 225 (39%) [66] | 110 (42.5%) [33] |
| Т3 [урТ3] | 27 (4.7%) [10] | 14 (5.4%) [10] |
| Incomplete data | 22 (3.8%) [17] | 13 (5%) \|5] |
| Total | 577 (100%) \|152] | 259 (100%) \|72] |
| **Tumour grade^b^** | | |
| Grade 1 | 50 (10.1%) | 26 (11.9%) |
| Grade 2 | 243 (49.2%) | 105 (48.2%) |
| Grade 3 | 197 (39.9%) | 83 (38.1%) |
| Incomplete/not determined | 4 (0.8%) | 4 (1.8%) |
| Total | 494 (100%) | 218 (100%) |
| **Hormone expression^c^** | | |
| ER positive | 437 (83.4%) | 200 (84.4%) |
| ER negative | 83 (15.8%) | 37 (15.6%) |
| Incomplete data | 4(0.8%) |  |
| Total | 524 (100%) | 237 (100%) |
| **HER-2 expression^b^** | | |
| HER-2 positive | 85 (17.2%) | 32 (14.7%) |
| HER-2 negative | 401 (81.2%) | 181 (83%) |
| Incomplete/not determined | 8 (1.6%) | 5 (2.3%) |
| Total | 494 (100%) | 218 (100%) |
| **Nodal metastasis^b^** | | |
| Node positive | 136 (27.5%) | 58 (26.6%) |
| Node negative | 353 (71.4%) | 157 (72%) |
| Incomplete | 5 (1%) | 3 (1.4%) |
| Total | 494 (100%) | 218 (100%) |
| **Focality** | | |
| Multifocal | 117 (20.3%) | 46 (17.8%) |
| Unifocal | 440 (76.2%) | 208 (80.3%) |
| Incomplete/not determined | 20 (3.5%) | 5 (1.9%) |
| Total | 577 (100%) | 259 (100%) |

^a^ With (non)invasive breast carcinoma; CPR = complete pathological response,

[ ] = number of patients received neo-adjuvant systemic treatment.

^b^ Invasive cancers only.

^c^ Hormone receptor expression was determined for 30 and 19 patients with DCIS,

respectively; n/a = not applicable.

**Supplementary Table 8.** Original Table 1 from Crown et al. (2021).

TABLE 1 Patient, tumor, and surgery characteristics

|  | Patients (n = 100) |
| --- | --- |
| *Patient characteristics* | |
| Age, years [mean (range)] | 59 (29-84) |
| *Tumor characteristics* | |
| Size on imaging, mm [mean (range)] |  |
| Unifocal tumors | 66.0 *±* 32.5 (50-160) |
| Span of multifocal/multicentric tumors | 59.4 ±24.5(35-130） |
| Size on final pathology, mm [mean] |  |
| Unifocal tumors | 78.7±30.1 |
| Largest single lesion of multifocal/multicentric tumors | 44.6±36.5 |
| Histology |  |
| Invasive ductal carcinoma | 73 |
| Invasive lobular carcinoma | 17 |
| Invasive carcinoma with ductal and lobular features | 10 |
| Grade |  |
| 1 | 13 |
| 2 | 50 |
| 3 | 37 |
| Focality |  |
| Unifocal | 19 |
| Multifocal/multicentric | 81 |
| Receptor profile | |
| ER/PR+, HER2- | 73 |
| ER/PR+, HER2+ | 13 |
| ER/PR-, HER2+ | 5 |
| Triple-negative | 9 |
| Presence of LVI | 31 |
| cN+ | 8 |
| pN+ | 19 |
| *Surgical techniques* |  |
| Reduction mammoplasty | 51 |
| Mastopexy | 25 |
| Racquet mammoplasty | 16 |
| Other | 8 |
| Contralateral symmetry procedure | 86 |
| *Surgical outcomes* |  |
| No tumor on ink | 80 |
| < 2 mm margins for DCIS | 34 |
| Attempt at re-excision | 45 |
| Completion mastectomy | 13 |
| *Axillary surgery* |  |
| Type of surgery |  |
| SNLB | 79 |
| ALND | 21 |
| Complications | 2 |
| *Adjuvant therapies* |  |
| Chemotherapy | 42 |
| Neoadjuvant chemotherapy | 8 |
| Adjuvant chemotherapy | 34 |
| Indications for chemotherapy | |
| cN+ | 8 |
| cN0 but pN+ | 19 |
| High-risk based on Oncotype DX RS | 8 |
| pN0 HER2+ > 5 mm | 6 |
| pN0 triple-negative > 5 mm | 1 |
| Whole-breast radiation (of 87 patients who achieved BCS) | 81 (93%) |
| Endocrine therapy (of 78 patients for whom endocrine therapy was recommended) | 75 (96%) |

ER estrogen receptor, PR progesterone receptor, LVI lymphovascular invasion, cN+ clinical axillary nodal disease, pN+ pathologically node-positive, DCIS ductal carcinoma in situ, OPS oncoplastic breast-con-serving surgery, SLNB sentinel lymph node biopsy, ALND axillary lymph node dissection, RS recurrence score, BCS breast-conserving surgery, NO clinically node-negative, pN0 pathologically node-negative

**Supplementary Table 9.** Original Table 1 from Falade et al. (2024).

TABLE 1 Patient characteristics and clinicopathologic features by BCS procedure type

|  | All  N=494 | Lumpectomy  n=326 | Lumpectomy with oncoplastic closure n=86 | ORM  n=82 | P value |
| --- | --- | --- | --- | --- | --- |
| Age, years^a^ | 61.4(12.0) | 62.5 (12.4) | 60.5(11.3) | 58.1 (10.4) | 0.01 |
| T stage^b^ |  |  |  |  | < 0.001 |
| 1 | 281 (57.5%) | 198 (61.5%) | 52(61.2%) | 31 (37.8%) |  |
| 2 | 153(31.3%) | 96(29.8%) | 26 (30.6%) | 31 (37.8%) |  |
| 3 | 55 (11.2%) | 28 (8.7%) | 7 (8.2%) | 20 (24.4%) |  |
| Tumor size, cm^b^ | 2.4(1.9) | 2.2(1.7) | 2.2(1.7) | 3.3 (2.5) | 0.001 |
| Lumpectomy volume, cm^3c^ | 94.0 (106.3) | 61.7(50.5) | 86.9 (76.8) | 189.5(170.1) | < 0.01 |
| Shave margins^d^ | 285(61.6%) | 157 (53.0%) | 61 (71.8%) | 67 (81.7%) | < 0.01 |
| N stage^b^ |  |  |  |  | 0.50 |
| 0 | 356 (72.8%) | 237 (73.8%) | 66 (76.7%) | 53 (64.6%) |  |
| 1 | 91 (18.6%) | 56(17.4%) | 15 (17.4%) | 20 (24.4%) |  |
| 2 | 25 (5.1%) | 15 (4.7%) | 4 (4.7%) | 6 (7.3%) |  |
| 3 | 17(3.5%) | 13 (4.1%) | 1(1.2%) | 3 (3.7%) |  |
| Tumor grade^c^ |  |  |  |  | 0.25 |
| 1 | 149(30.8%) | 108(34.1%) | 20 (23.5%) | 21 (25.9%) |  |
| 2 | 312(64.6%) | 195(61.5%) | 62 (72.9%) | 55 (67.9%) |  |
| 3 | 22(4.6%) | 14 (4.4%) | 3 (3.5%) | 5 (6.2%) |  |
| Tumor receptor subtype^f^ |  |  |  |  | 0.56 |
| ER+PR+HER- | 365 (79.7%) | 231 (79.1%) | 70 (82.4%) | 64 (79.0%) |  |
| ER+PR-HER- | 55 (12.0%) | 33(11.3%) | 11(12.9%) | 11 (13.6%) |  |
| ER-PR-HER- | 10(2.2%) | 9 (3.1%) | 1(1.2%) | 0 (0%) |  |
| HER2+ | 28 (6.1%) | 19 (6.5%) | 3 (3.5%) | 6 (7.4%) |  |
| Tumor multifocality present^g^ | 148 (30.7%) | 98 (30.9%) | 21 (24.4%) | 29 (25.8%) | 0.32 |
| Positive margin rate^h^ | 186(38.3%) | 132(41.4%) | 28 (32.9%) | 26 (31.7%) | 0.15 |
| Follow-up time (years)^a^ | 8.0 (6.5) | 9.9 (6.9) | 4.1 (3.5) | 4.6 (3.6) | < 0.001 |

*BCS* breast-conserving surgery; *ORM* oncoplastic reduction mammoplasty; *ER* estrogen receptor; *PR* progesterone receptor; *HER2* human epidermal growth factor receptor 2

^a^ Data available in 494 cases

^b^ Data available in 489 cases

^c^ Data available for 368 cases

^d^ Data available for 463 cases

^e^ Data available in 483 cases

^f^ Data available in 453 cases

^g^ Data available in 482 cases

^h^ Data available in 486 cases
